# Prediction of lung function using handgrip strength in healthy young adults

**DOI:** 10.14814/phy2.13960

**Published:** 2019-01-10

**Authors:** Nnamdi C. Mgbemena, Happiness A. Aweto, Bosede A. Tella, Theophilus I. Emeto, Bunmi S. Malau‐Aduli

**Affiliations:** ^1^ Discipline of Physiotherapy College of Healthcare Sciences James Cook University Townsville Queensland Australia; ^2^ Physiotherapy Department University of Lagos, Idi‐ Araba Lagos Lagos State Nigeria; ^3^ Public Health & Tropical Medicine College of Public Health Medical and Veterinary Sciences James Cook University Townsville Queensland Australia; ^4^ College of Medicine and Dentistry James Cook University Townsville Queensland Australia

**Keywords:** Handgrip strength, lung function, prediction

## Abstract

Positive association between physical activity and spirometry has been reported to be possibly attributed to handgrip strength (HGS), particularly in the elderly. However, the nature of the association between HGS and lung function in young adults is still unclear. This study investigated the prediction of lung function using HGS in young adults. A cross‐sectional analytical study was carried out on four hundred (400) apparently healthy medical students who are aged 16–30 years. Handgrip strength (dominant and nondominant) and lung function (FEV_1_, FVC and PEFR) of these students were assessed using Jamar dynamometer and a portable spirometer, respectively. Data were analyzed using inferential statistics. Independent *t*‐test showed that the mean values of HGS and lung function of the males were significantly higher than the females (*P* < 0.0005). The relationship between HGS and lung function indices was significant (*P* < 0.0005) in all the participants but strongest for FEV_1_ (*r* = 0.64). The regression analysis showed that in addition to gender and height, HGS was a significant (*P* < 0.0005) predictor of lung function. Regression equations were also proposed for the prediction of these lung function indices using HGS, gender and height. This study is the first to report HGS as a significant predictor of pulmonary function in healthy young adults living in a low‐resource country. Hence, its use could enhance medical practice in being an indicator of lung function status in healthy young adults.

## Introduction

Handgrip strength (HGS) is the force produced due to joint activities of the deep‐seated and superficial hand and forearm muscles during gripping (Koley and Kumaar 2011). It is an inexpensive, noninvasive and objective indicator of an individual's health status and muscle strength (Ortega et al. 2012). Studies have reported that it can be used to monitor nutritional intervention in healthy young adults (Norman et al. 2010), predict physical function in people living with HIV/AIDS (Raso et al. 2013) and differentiate the presence or absence and severity of asthma in children (Latorre‐Román et al. 2014). Additionally, reference values of HGS have been suggested to be applicable in evaluating the level of recovery in patients with functional impairment of upper extremities (Adedoyin et al. 2009). Furthermore, HGS has been recommended as a relevant instrument in health and nutritional evaluation in students where body mass index (BMI) was one of its determining factors and in antenatal care considering its prognostic advantages (Ibegbu et al. 2014; Mbada et al. 2015; Hammed and Agbonlahor 2017). Factors like age, gender, height, weight, ethnicity, nutritional status and levels of physical activity have been reported to influence handgrip strength (Adedoyin et al. 2009; Kubota and Demura 2011; Koopman et al. 2015; Manoharan et al. 2015).

Low to middle‐resource countries (LMRC) have reported physical activity (PA) levels lower than the World Health Organisation (WHO) recommendations (Smith et al. 2016). LMRC have also been characterized by increased effect of noncommunicable lung diseases such as bronchial asthma and chronic obstructive pulmonary diseases (COPD) which account for >90% of deaths in such settings (Beran et al. 2015). Nonetheless, higher levels of PA have been reported to be associated with improved lung function in healthy adults (Luzak et al. 2017). The Global Initiative for Chronic Obstructive Lung Disease (GOLD) (2018) has approved spirometry as a noninvasive tool used for lung function tests, that is, in evaluating the respiratory status of an individual (Fawibe et al. 2017). These tests (spirometric indices) are (i) forced expiratory flow in 1 sec (FEV_1_), (ii) forced vital capacity (FVC) and (iii) peak expiratory flow rate (PEFR); they involve forceful exhalation of air from the lungs and they have become standard practices done during health examination in occupational health assessment and sports sciences (Ferguson et al. 2000). Interpretation of these lung function indices is commonly expressed as percentage of predicted (%Pred) which involves comparing the observed lung function values with predicted values based on an individual's height, age and gender (Pakhale et al. 2009). In 2012, the Global Lung Function Initiative (GLI) developed prediction models for lung function from four ethnic groups excluding African groups (Culver et al. 2017). However, results from recent study by Arigliani et al. (2017), have supported the applicability of GLI‐2012 reference values for African Americans in predicting spirometric values for sub‐Saharan Africans. Furthermore, use of these spirometric indices are still under‐utilized in LMRC particularly due to its high cost and inadequate training in lung function testing for health professionals (Desalu et al. 2010; Grigsby et al. 2016).

Studies have reported that the positive association between PA and spirometric indices may be attributed to some extent by muscle strength which may explain the link between spirometric indices and HGS (Nystad et al. 2006; Berntsen et al. 2008; Smith et al. 2018). Most studies are reported on the relations between HGS and spirometric indices in the elderly (Holmes et al. 2017; Son et al. 2018). However, to our knowledge, studies reported in healthy young adults are still lacking. Henceforth, the nature of the association between HGS and lung function is still uncertain in healthy young adults. Early identification of changes in pulmonary function with the aid of noninvasive, inexpensive and easily assessed HGS would be of practical benefit, particularly in LMRC, if these were identifiable early in adult life. Therefore, in this study, we aimed to examine the relationship between HGS and lung function in healthy young adults. We also investigated the predictability of lung function indices using HGS and not just anthropometric parameters.

## Methods

### Ethical approval

Ethical approval was sought and obtained from the Lagos University Teaching Hospital Health Research Ethics Committee (Assigned No: ADM/DCST/HREC/APP/728), Idi‐Araba, Lagos.

### Participants

Participants included apparently healthy young adults aged between 16 and 30 years, who were undergraduate students of the College of Medicine, University of Lagos (CMUL), Idi Araba, Lagos, Nigeria. The CMUL has a population of over 2339 students and currently made up of three faculties. Participation was voluntary and informed consent was obtained from participants prior to commencement of the study. Students who had the following issues were excluded from the study: visible limitations in either hand, surgery in the hand or wrists in the last 3 months, obesity, asthma, history of a respiratory disease, an existing or a history of cardiovascular disease or cigarette smokers.

### Study design and sampling technique

This study employed a cross‐sectional analytical design. A multistage sampling technique was used to recruit the participants. Computer‐generated numbers were used to obtain two faculties out of the three faculties in College of Medicine. From these two faculties, two departments each (with four departments in total) were selected using the computer generated numbers. Still using electronic numbers, two levels of study was obtained from each of the four departments (with eight levels in total). Finally, fifty students (25 males and 25 females) were obtained electronically from each level of study in each department using their class list. Altogether, four hundred (400) students were involved in the study.

### Procedure

Sociodemographic parameters like age, gender, weight, height, and BMI were obtained from participants at the start of the study, using a short questionnaire.

### Lung function assessment

The portable spirometer (Contec SP10, China) was used to measure the FEV_1_, FVC, and PEFR. A disposable mouthpiece was used for each participant. The participant inhaled maximally through the nose until the lungs were full. Afterward, the participant placed the spirometer through the disposable mouthpiece in his/her mouth, with lips sealed tightly around the mouthpiece while holding the lungs full (Johns and Pierce 2008). The participant was instructed to exhale forcefully as long as possible into the spirometer until no air could be exhaled (Queensland Health, 2012). This was done for a minimum of three trials as the FEV_1_, FVC, and PEFR values were obtained.

It was ensured that repeatability criterion was considered. This means that for the FEV_1_, the two highest values were within 0.150 L of each other. The two highest values of FVC were also within 0.150 L of each other. For FEV_1_ and FVC, the higher value between the two repeatable values was the accepted value. The highest value of PEFR was the accepted value (Johns and Pierce 2008; Queensland Health, 2012). Percentage predicted FEV_1_ and FVC were estimated using the prediction model for African‐American ethnic groups proposed by GLI‐2012 (Quanjer et al. 2012; Arigliani et al. 2017). This calculation was done using a software (Microsoft Excel sheet) developed by Sanja Stanojevic (https://www.ers-education.org/guidelines/global-lung-function-initiative/spirometry-tools/excel-sheet-calculator.aspx) that required height, age, gender, FEV_1_ and FVC actual values of the participants.

### Handgrip strength assessment

The Jamar dynamometer (Model J00105, USA) was used to measure handgrip strength. The participants’ hand dominance was recorded as participants sat comfortably on a seat without an armrest, with the shoulders adducted to the side, the elbow was in 90° flexion, and the forearm and wrist were in neutral position. The dynamometer metal clip was set at the second handle position in the lower arm of the dynamometer (Bae et al. 2015). Standardized instructions were adopted and used as suggested by the American Society of Hand Therapists (ASHT) (Adedoyin et al. 2009). It was ensured that the squeeze phase did not last more than 6 sec and an average of three readings were obtained for both hands (Sindhu et al. 2012). The average of the three readings for each of the two hands was calculated for each participant and recorded.

### Data analysis

Analysis of deidentified data was conducted using SPSS version 25.0 (IBM, Chicago, IL, USA). Anthropometric characteristics of the participants were presented using mean and standard deviation as data met the assumption for normality. Differences between the lung function indices, anthropometric parameters, HGS by gender were compared using the Independent Samples *t*‐test. Paired *t*‐test was used to compare the mean values between the dominant and the nondominant hands of the male and female participants. Pearson correlation was employed to determine the strength of the relationship between the handgrip strength (dominant and nondominant) and lung functions (FEV_1_, %Pred FEV_1_, FVC, %Pred FVC and PEFR).

Multiple regression analysis was used to determine the predictive values of lung function indices (outcome variables) using HGS, with age, gender, height and weight as co‐variates. Assumptions of linearity, independence of errors, homoscedasticity, unusual points and normality of residuals were met. All statistical tests were compared using a two‐tailed comparison with 95% level of confidence.

## Results

### Anthropometric characteristics

Four hundred (400) healthy young adults (undergraduates) were involved in the study with two hundred (200) male and female participants. The minimum and maximum values for age, height, and weight of the participants were 17 and 30 years; 1.49 and 2.01 m; 41 and 112 kg, respectively. There were significant differences in age, height and weight (*P* < 0.0005) as the male participants had higher mean values than the females (Table 1). Male participants also had higher mean BMI scores than their female counterparts but the difference was not statistically significant.

**Table 1:** Anthropometric characteristics of the participants

Variables	Males (*n* = 200)	Females (*n* = 200)	All (*n *=* *400)	*t* (df)	*P*‐value
	Mean (SD)	Mean (SD)	Mean (SD)		
Age (years)	21.69 (2.73)	20.46 (2.13)	21.07 (2.52)	5.050 (375.609)	<0.0005
Height (m)	1.76 (0.76)	1.65 (0.67)	1.70 (0.09)	14.605 (398)	<0.0005
Weight (kg)	70.34 (10.70)	61.40 (10.23)	65.87 (11.37)	8.535 (398)	<0.0005
BMI (kg/m^2^)	22.75 (2.55)	22.43 (2.85)	22.59 (2.70)	1.180 (393.913)	0.239
FEV_1_ (L)	3.36 (0.57)	2.38 (0.36)	2.87 (0.68)	20.635 (336.865)	<0.0005
FVC (L)	3.73 (0.82)	2.61 (0.42)	3.17 (0.86)	17.327 (297.191)	<0.0005
PEFR (L/sec)	7.71 (1.77)	5.60 (1.37)	6.66 (1.90)	13.350 (374.397)	<0.0005
DHGS (kgf)	39.88 (8.40)	26.12 (5.70)	32.21 (9.61)	19.159 (350.189)	<0.0005
NDHGS (kgf)	35.95 (8.10)	22.91 (5.35)	30.21 (10.02)	19.005 (345.263)	<0.0005
*t* (df)	16.707 (199)	20.277 (199)			
*P*‐value	<0.0005	<0.0005			

BMI, body mass index; *t*,* t* value; df, degree of freedom; *P*, significance level; FEV_1_, forced expiratory volume in 1 sec; FVC, forced vital capacity; PEFR, peak expiratory flow rate; DHGS, dominant hand grip strength; NDHGS, nondominant hand grip strength; SD, standard deviation; kgf, kilogram force.

### Influence of gender on lung function and handgrip strength

The independent *t*‐test showed that the mean FEV_1_ (3.36 ± 0.57), FVC (3.73 ± 0.82) and PEFR (7.71 ± 1.77) values were significantly higher for males compared to females (*t* = 20.635; 17.327; 13.350, respectively; *P* < 0.0005) (Table 1). Similarly, assessment of HGS suggest that the dominant handgrip strength (DHGS, 39.88 ± 8.40 kgf) and nondominant handgrip strength (NDHGS, 35.95 ± 8.10 kgf) for males were significantly higher than females (*t* = 19.159 and 19.005, respectively). Paired *t*‐test analysis also showed that the DHGS was significantly higher than the NDHGS in both males and females participants (*t* = 16.707 and 20.277, respectively) (Table 1).

### Relationship between handgrip strength and lung function

Pearson correlation analysis showed that FEV_1_ had the strongest significant correlation (*r* = 0.64, 0.63, respectively; *P* < 0.0005) with both DHGS and NDHGS for all participants. This was followed by the FVC and PEFR which were also significantly correlated with both DHGS and NDHGS for all participants (*r* = 0.49; 0.61 and 0.51, respectively). Likewise, there were statistically significant moderate (%Pred FEV_1_) and small (%Pred FVC) correlations with HGS, respectively, *P* < 0.0005 (Table 2).

**Table 2:** Correlation between handgrip strength and lung function

Variables		FEV_1_	%Pred FEV_1_	FVC	%Pred FVC	PEFR
DHGS	*r*	0.64	0.34	0.61	0.27	0.51
	*P*	<0.0005	<0.0005	<0.0005	<0.0005	<0.0005
NDHGS	*r*	0.63	0.34	0.61	0.29	0.49
	*P*	<0.0005	<0.0005	<0.0005	<0.0005	<0.0005

FEV_1_, forced expiratory volume in 1 sec; %Pred FEV_1_, percentage predicted FEV_1_; FVC, forced vital capacity; %Pred FVC, percentage predicted FVC; PEFR, peak expiratory flow rate; DHGS, dominant handgrip strength; NDHGS, nondominant handgrip strength; *r*, Pearson's correlation coefficient; *P*, significance level.

### Prediction of lung function using handgrip strength

We ran a series of multiple regression analyses to predict the lung function indices (FEV_1_, FVC and PEFR) from DHGS or NDHGS and age, gender, weight, and height.

The multiple regression models using DHGS and NDHGS statistically significantly predicted the following: (i) FEV_1_ (*F* (5, 394) = 149.846, *P *<* *0.0005, adj. *R* ^*2*^ = 0.66 and *F* (5, 394) = 148.621, *P *<* *0.0005, adj. *R* ^*2*^ **= 0.65, respectively); (ii) FVC (*F*(5, 394) = 104,561, *P *<* *0.0005, adj. *R* ^*2*^ = 0.57 and *F* (5, 394) = 105.745, *P *<* *0.0005, adj. *R* ^*2*^ = 0.57, respectively) and (iii) PEFR (*F*(5, 394) = 49.618, *P *<* *0.0005, *R* ^*2*^ = 0.38 and *F* (5, 394) = 47.919, *P *< 0.0005, *R* ^2^ = 0.37, respectively).

Gender, height and handgrip strength added statistically significantly to the prediction models for all lung functions variables assessed (*P *<* *0.0005). The age and weight of the participants had negative and positive coefficients respectively, in all the prediction models (Table 3).

**Table 3:** Regression variables for the lung function using handgrip strength and other co‐variates

Dominant handgrip strength							Nondominant handgrip strength					
Variables	Intercept	DHGS	Gender	Height	Age	Weight	Intercept	NDHGS	Gender	Height	Weight	Age
FEV_1_												
* β*	−2.467	0.013	0.497	2.703	−0.008	0.003	−2.498	0.013	0.503	2.743	−0.008	0.004
SE_B_	0.565	0.003	0.061	0.373	0.009	0.003	0.567	0.003	0.062	0.373	0.009	0.003
*B*		0.191	0.365	0.351	−0.031	0.057		0.179	0.370	0.356	−0.030	0.059
*P*	0.000	0.000	0.000	0.000	0.350	0.186	0.000	0.000	0.000	0.000	0.348	0.156
FVC												
*β*	−3.403	0.019	0.492	3.365	−0.013	0.003	−3.454	0.021	0.476	3.420	−0.013	0.003
SE_B_	0.795	0.004	0.086	0.524	0.012	0.004	0.792	0.004	0.086	0.522	0.012	0.004
*B*		0.225	0.287	0.347	−0.037	0.044		0.236	0.278	0.352	−0.039	0.042
*P*	0.000	0.000	0.000	0.000	0.316	0.356	0.000	0.000	0.000	0.000	0.272	0.343
PEFR												
*β*	−3.898	0.041	1.012	5.429	−0.027	0.001	−3.984	0.033	1.109	5.560	−0.023	0.002
SE_B_	2.099	0.011	0.227	1.383	0.032	0.010	2.113	0.012	0.229	1.392	0.032	0.010
*B*		0.212	0.267	0.253	−0.036	0.004		0.164	0.293	0.259	−0.031	0.012
*P*	0.059	0.001	0.000	0.000	0.411	0.945	0.065	0.004	0.000	0.000	0.473	0.810

DHGS, dominant handgrip strength; FEV_1_, forced expiratory volume in 1 sec; FVC, forced vital capacity; PEFR, peak expiratory flow rate; *β*, unstandardized coefficient; SE_B_, standard error of the coefficient; B, standardized coefficient; *P*, significance level.

We generated the following regression equations proposed for predicting the lung function indices (note the reference group for gender is females, Table 3).

For prediction using DHGS:

$${\text{FEV}}_{1}.=013\left(\text{HGS}\right)+2.703\left(H\right)+0.497\left(G\right)+0.003\left(W\right)-0.008\left(A\right)-2.467$$

FVC=0.019(HGS)+3.365(H)+0.492(G)+0.003(W)−0.013(A)−3.403

PEFR=0.041(HGS)+5.429(H)+1.012(G)+0.001(W)−0.027(A)−3.898

For prediction using NDGHS:

$${\text{FEV}}_{1}=0.013\left(HGS\right)+2.743\left(H\right)+0.503\left(G\right)+0.004\left(W\right)-0.008\left(A\right)-2.498$$

FVC=0.021(HGS)+3.420(H)+0.476(G)+0.003(W)−0.013(A)−3.420

PEFR=0.033(HGS)+5.560(H)+1.109(G)+0.002(W)−0.023(A)−3.984,

(where HGS = handgrip strength, H = height, G = gender, W = weight, and A = age). For DGHS, the predicted FEV_1_, FVC, and PEFR for males is 0.497, 0.492, and 1.012 greater than that predicted for females, respectively (with all other independent variables held constant). This is similar for NDGHS.

## Discussion

This study was carried out to investigate the prediction of lung function indices (FEV_1_, FVC, and PEFR) using HGS (dominant and nondominant) in healthy young Nigerian adults. The results showed that the mean values of HGS and lung function indices in males are higher than in females. There was a significant positive relationship between HGS and lung function indices of the participants. Regression equations were proposed as HGS was among the significant predictors of lung function in this study. To the best of our knowledge, no study has investigated the ability of HGS to predict lung function status in healthy young adults from low resource countries (LRC). The results from this study have demonstrated that in LRC settings, where it may be difficult to afford sufficient equipment (spirometers) for lung function assessment, reference equations involving the use of a simple and easily assessable tool like HGS, can be employed to predict lung function indices without relying on anthropometric parameters. It is hoped that the lung function data from this study could be a valuable addition to the existing Global Lung Initiative database of normative values from LMRC.

The observed higher mean height and weight values for males in comparison to their female counterparts corroborate previous studies done in other LRC (Knudsen et al. 2011; Musafiri et al. 2013; Fawibe et al. 2017). This finding may be attributed to hormonal effects between both genders which translate to having longer bones and increased muscle mass in males than in females whose bony epiphyseal plates close at an early age (Ogunlade and Adalumo 2015). Similarly, the BMI of the male participants was higher, though this was not significantly different to that of the females. The nonsignificant BMI values may be attributed to the apparently healthy state and smaller age range of the participants included in this study.

The observed significantly higher HGS in males than in females corroborates previous studies done in similar populations (Balogun et al. 1991; Adedoyin et al. 2009; Michael et al. 2013; Ibegbu et al. 2014) and internationally (Moy et al. 2015; Ro et al. 2015; Vivas‐Diaz et al. 2016; Holmes et al. 2017). This could be as a result of hormonal influences as previously mentioned which enhances longer bone and muscle growth, thereby encouraging greater muscle contractile units (Balogun et al. 1991) and the increased involvement of men in leisure time activities than women (Aadahl et al. 2011). Furthermore, Kulaksiz and Gözil (2002) in their study, reported that in young adults, males have longer and “square‐ shaped” hands which correlates with their height than in their female counterparts. The significant difference between the DHGS and NDGHS within gender could be explained by constant use of the dominant hand in performing various daily tasks (Kubota and Demura 2011).

Evaluation of the lung function indices suggested males had significantly higher mean values than females. This result was expected as the male participants were taller than females and previous studies have reported height as a strong predictor of lung function (Nku et al. 2010; Fawibe et al. 2017). This will translate to having larger intrathoracic space for increased lung expansion and higher volumes. This result was also consistent with the findings in other developing and developed countries (Knudsen et al. 2011; Musafiri et al. 2013; Smith et al. 2018). Fawibe et al. (2017) reported lower mean lung function values than this present study and this may be as a result of the older population included (56–65 years) in their study which would negatively affect the lung function values as a result of increasing age.

The lung function parameters assessed were shown to be significantly associated with the HGS of the participants. This corroborates previous findings (Rozek‐Piechura et al. 2014; Bae et al. 2015; Holmes et al. 2017; Smith et al. 2018; Son et al. 2018) and could be explained by the strong relationship reported between skeletal muscle strength and respiratory muscle strength, particularly, the Maximal inspiratory pressure (MIP) of the diaphragm (Shin et al. 2017). Therefore, a reduced MIP translates to lower lung functions in an individual and could inform an impairment in the lungs. (Bahat et al. 2014). The moderate to high correlation between handgrip strength and lung function reported in this study could be an indicator of a healthy state of the participants’ respiratory systems. Furthermore, previous study showed that handgrip strength usually attains its apex at ages 21–30 (Adedoyin et al. 2009) with FEV_1_ and FVC increasing in a steady rate from birth until age 25. These lung function parameters usually assume a plateau phase for 5–10 years before decreasing as an individual gets older (Ostrowski and Barud 2006). Interestingly, the FEV_1_ and FVC had stronger correlations with HGS than PEFR in our study and this could be due to the age range of our participants falling within these peak periods. Conversely, a study by Bahat et al. (2014) reported that there was no association between HGS and lung function in older males living in nursing homes. The dissimilarity could be attributed to factors like smaller sample size, increased age and high sedentary state of their participants. The moderate and small moderate correlations between the HGS and %Pred lung function (FEV_1_ and FVC) could be attributed to the use of the prediction model of GLI African‐American ethnic group in calculating these percentages. Despite the good fit that may be expected between African‐American and African populations, factors like genetic mixing, higher socioeconomic and nutritional status which influence lung function observed in African American groups could contribute to the reported relationship (Glew et al. 2004; Arigliani et al. 2017).

The regression equations from our study demonstrated height, gender and HGS as the significant predictors of lung function, while excluding age and weight. This echoed previous studies where only height and age were independent predictors in both male and female participants (Hankinson et al. 1999; Knudsen et al. 2011; Musafiri et al. 2013; Fawibe et al. 2017).

The narrow age range of (16–30 years) of the participants in this study may have limited the generalisability of our findings to other LRC settings. Additionally, the participant group selected for this study were well‐informed medical students who were aware of the effects of overweight and the importance of maintaining good health habits. This choice of participants could have also influenced our findings. Furthermore, factors such as physical activity and ethnicity that influence lung function were not considered in this study. Future studies could involve diverse participant groups with wider age ranges, and assessment of factors such as physical activity levels to further examine the relationship between HGS and lung function. Overall, the practical implications and benefits of this study far outweigh its limitations. The study is the first to report HGS as a significant predictor of lung function in an LRC. It gives a groundwork indication in estimating the lung function of healthy young adults using an objective and simpler test like handgrip strength and not just with the use of anthropometric measurements.

## Conclusion

Handgrip strength is associated with lung function and more specifically, the FEV_1_ and FVC, which measure the size of the lungs. Grip strength is also a significant predictor of pulmonary function in healthy young adults living in a low‐resource country. Hence, utilization of noninvasive, inexpensive and simple handgrip strength test in low to middle‐resource countries could enhance medical practice in being an indicator of lung function status in a healthy young adult.

## Conflicts of Interest

The authors declare that they have no conflicts of interest.

## Conflict of Interest

None declared.
